# Fracture risk assessment by dual-energy absorptiometry in osteogenesis imperfecta: A systematic review of measurement sites and its predictive value

**DOI:** 10.1016/j.bonr.2026.101929

**Published:** 2026-06-10

**Authors:** Karlijn Scheepens, Suzanne den Haan, Kelly Warmink, Ralph Sakkers, Anne Spaans, Wouter Nijhuis, Harrie Weinans

**Affiliations:** a University Medical Centre Utrecht, Department of Orthopaedics, Heidelberglaan 100, 3584 CX, Utrecht, the Netherlands

**Keywords:** Osteogenesis imperfecta, Fracture risk prediction, Dual-energy absorptiometry

## Abstract

People with osteogenesis imperfecta (OI) suffer frequent fracturing of bones. Dual-energy absorptiometry (DXA) is considered a surrogate measure of bone strength, and DXA scans are usually acquired yearly to guide treatment. However, DXA provides only limited insight into bone strength as it does not account for bone geometry or microarchitecture. This systematic review outlines current clinical practice regarding the DXA in children with OI, and evaluates evidence for conventional and alternative DXA-derived measures in OI. The search was conducted in Pubmed, Embase, and Web of Science databases at 12-Dec-2025 and records were screened by two reviewers. Studies presenting DXA measurement locations in children with OI or results on the relation between DXA-derived measures and OI type or fractures were eligible and presented in a descriptive manner. Risk of bias was assessed using the Newcastle-Ottawa Scale. In total, 91 studies were included. Data was extracted from 38 observational studies including 3696 patients. The results showed that DXA measurement locations are not standardized. Although studies were variable in methods and results, the association between DXA-derived areal bone mineral density (aBMD) and fractures was generally weak – particularly when fracture incidence was evaluated over short time intervals. However, location-specific measurements may offer some improvement. DXA-based measures reflecting trabecular structure or estimated bone volume did not outperform aBMD. Given the presented results of conventional DXA in OI, alternative methods of bone strength assessment in patients with OI are needed. Assessment targeting fracture-prone skeletal regions and incorporating geometric properties may enhance DXA's predictive value.

## Highlights

• DXA measurement locations are not standardized for osteogenesis imperfecta (OI).
• Areal bone mineral density has limited utility for fracture risk prediction in OI.
• DXA-derived TBS and BMAD show no association with fracture risk in OI.
• Diaphyseal DXA incorporating bone geometry may improve clinical assessment in OI.

## 1 Introduction

In the heterogeneous osteogenesis imperfecta (OI) population, an inherited genetic defect in one of the genes related to bone formation leads to bone fragility and deformities. OI patients are clinically classified in type I to V based on their functioning and radiological characteristics (Van Dijk and Sillence, 2014). While type II is perinatally lethal, type III is the most severe patient group that generally uses a wheelchair for mobility, type I is relatively mild and type IV is moderate. OI type V features interosseous membrane calcification and a tendency toward hyperplastic callus formation. In children, the three important treatment pillars are rehabilitation therapy, pharmaceutical treatment with bisphosphonates (BP), and surgical treatment for straightening and stabilization of bones (Cho et al., 2020; Engelbert et al., 2004; Fassier, 2021; Glorieux et al., 1998). Children with OI are usually monitored yearly using Dual-energy X-ray absorptiometry (DXA) in order to guide their treatment (Liu et al., 2023). There is no consensus concerning the exact measurement locations and many different DXA protocols and evaluation criteria are being used for OI children.

DXA is an imaging modality based on X-ray radiation which quantifies the amount of mineral in bone, making it applicable to diseases that are characterized by compromised bone health (Choksi et al., 2018). Since DXA is two-dimensional, the bone mass is quantified per bone area within the projection (areal bone mineral density (aBMD)) in g/cm^2^ (Berger, 2002). As aBMD increases with growth, it is compared to a healthy population of the same age and sex to calculate the *Z*-score, which represents the difference in standard deviations from this age-matched population mean (Zemel et al., 2011).

The traditional target locations lumbar spine and proximal femur are chosen based on their high incidence of fracture in the elderly osteoporosis population, for which this technique was first introduced (Choksi et al., 2018). For all children, the International Society For Clinical Densitometry recommends bone density measurement at the lumbar spine and the total body excluding head. Other sites can be used depending on the individual case (International Society For Clinical Densitometry, 2019). To measure the density at a specific location, the manufacturer software automatically selects the contours of the bone within the area of interest. All pixel intensities representing mineral mass in grams within the contours are summed. This is divided by the area of all pixels within the contours to calculate the aBMD (Berger, 2002).

However, DXA outcomes give only very limited insight in bone strength. Several determinants of bone strength are not differentiated in DXA assessment (Choksi et al., 2018). First of all, bone geometry is not taken into account in the results, while it has a significant influence on the measured density. In case of identical volumetric bone density, smaller bones will show a lower aBMD than larger bones (Binkovitz et al., 2008) and wider shaft bones will usually resist more bending forces than smaller shaft bones (Hart et al., 2017). Bowing deformities cause stress accumulation in the shaft and thereby increased risk of fracture. Secondly, the trabecular and cortical components of bone architecture have distinct properties, each with a unique role for bone strength, but DXA cannot distinguish between these compartments.

In addition to the limitations of the technique, some patient-related factors make fracture prediction with DXA particularly difficult in OI children. Deformities and (healing) fractures influence the area of the bone that is assessed and therefore the measured density. Moreover, metal rods and plates used for deformity correction and fracture stabilization create artefacts in the DXA data and increase the resulting aBMD (Weaver et al., 2021).

Despite the limitations of the technique, the usefulness of DXA for osteoporosis is widely accepted. A large meta-analysis has shown that DXA has predictive value for osteoporotic fractures at several locations and in many age categories in elderly populations (Marshall et al., 1996). In children with OI, the predictive value of DXA is less well-established. In 1994, even before the introduction of BP that markedly increase bone density (Sakkers et al., 2004), Patterson et al. recruited 58 type I and type IV OI patients for forearm bone density measurements and concluded that ‘bone density in OI may well be normal’ (Paterson and Mole, 1994).

Improving bone strength assessment for OI is an active topic of research. Several studies have tried to extract parameters from standard DXA scans that may improve the fracture prediction capacity, for example the Trabecular Bone Score (TBS). This systematic review outlines the current clinical practice of DXA for fracture risk assessment in children with OI, and evaluates the supporting evidence for aBMD and alternative parameters derived from DXA.

## 2 Methods

This systematic review is structured in four sections describing 1) DXA measurement locations, 2) differences in DXA outcomes between OI types, 3) the association of DXA results with fracture risk, and 4) parameters from DXA besides aBMD.

### 2.1 Literature search

The PubMed, Embase and Web of Science databases were searched at 14-Feb-2025 using the following terms and all related entries: [(Bone density OR DXA) AND Osteogenesis Imperfecta]. Inclusion of the term ‘bone strength’ did not lead to extra relevant publications and was therefore left out. Duplicates were removed. A second search was performed at 12-Dec-2025 to include relevant papers published over the last ten months. Two reviewers independently reviewed first the title/abstract and if necessary the full text of all search results. In case of disagreements, discussion led to consensus. Additionally, references were screened by one of the authors and included if relevant for this review. No automation tools were used for record screening or data extraction.

### 2.2 Eligibility criteria

In order to be included, the publications needed to 1) present original research, 2) concern OI patients and 3) present DXA-derived measures of bone density, bone quality or bone strength. The studies needed to describe a) the DXA measurement locations in their medical centre, or b) the relation of DXA-derived aBMD or an alternative DXA-derived parameter to clinical OI type or fractures. Regarding subject a), only studies presenting at least ten paediatric OI patients were included. Regarding subject b), studies describing only adult OI patients were also included to increase the amount of available data. Papers written in other languages than English were excluded and the full-text articles needed to be available for the reviewers.

### 2.3 Data extraction process

Following screening, the papers were allocated to one of the four sections described below based on the presented data, and data extraction was performed by one of the authors.

#### 2.3.1 DXA measurement locations

The global area of interest (e.g. lumbar spine) and the medical centre where the research was located were extracted from the papers. If available, the specific location (e.g. L1-L4) was included. For each research site, only the most recent publication presenting the DXA location in OI children was included. If an older paper from the same research site described more locations than the most recent, this paper was also included.

#### 2.3.2 Differences in DXA outcomes between OI types

Studies in adults as well as in children comparing at least two types of OI were included. OI types were based on clinical assessment according to Sillence (Sillence and Rimoin, 1978), since studies published before the clinical categorization refinement by Van Dijk et al. (Van Dijk and Sillence, 2014) were also eligible for this review. In case of a longitudinal study design, only the first DXA assessment was included in this review. To make the DXA well comparable across patient cohorts of varying ages, only the studies presenting *Z*-scores were included. Given the heterogeneity of the studies, results were descriptively presented and an unweighted mean was calculated per OI type to provide an overview. If necessary, calculations were performed enabling to present the data in a standardized way. For example, the weighted average was calculated if data were presented for males and females separately. If statistical testing was performed, the results were presented. The number of subjects, their mean age, and the use of BP within the study population were furthermore extracted.

#### 2.3.3 The association of DXA with fracture risk

From studies including aBMD and/or Z-scores from any DXA location, a fracture endpoint and a statistical analysis of the association between these parameters, the effect measures and effect sizes were included. Studies were not restricted based on the type of effect measures reported. Moreover, the type of study, number of subjects, their clinical types, mean age, and treatment history with BP – if available – were extracted. Given the substantial methodological heterogeneity, the association between DXA outcomes and fracture risk was summarized descriptively, and no meta-analysis was done.

#### 2.3.4 Parameters from DXA besides aBMD

Studies performing an extra analysis on standard DXA data from any location with the goal to improve bone strength assessment and presenting results in OI patients were included to this section. Studies applying automized software for vertebral fracture detection on lateral spine DXA scans (dual-energy vertebral fracture assessment (VFA)) were not included in our review, since this is considered a fracture detection rather than a fracture risk estimation method. The derivation of the DXA-based parameters, and – if available – the type of study, number of subjects, their clinical types, mean age, and treatment history with BP were included. If the association with a fracture outcome was assessed, all presented effect measures, the statistical methodology, and effect sizes were included in this review. These results were only descriptively presented because of the limited amount of data.

### 2.4 Risk of bias assessment

Risk of bias was assessed for all studies on differences in DXA outcomes between OI types, associations between DXA results and fracture risk, and DXA-derived parameters other than aBMD. The Newcastle-Ottawa Scale (NOS) was used for cohort and case-control studies. This tool evaluates methodological quality across three domains: selection of study groups, comparability of groups, and ascertainment of exposure for case-control studies or outcome for cohort studies.

For cross-sectional studies, the adapted Newcastle-Ottawa Scale for cross-sectional studies (NOS-xs), developed by Carra et al. (2025), was used. The NOS-xs evaluates studies across the domains of study sample, exposure and outcome assessment, and adjustment for confounders. Because only baseline or first available DXA measurements were extracted from longitudinal studies in the section on differences in DXA outcomes between OI types, these studies were assessed as cross-sectional studies using the NOS-xs.

Studies could receive a maximum of nine stars, with scores of 0–3 stars indicating high risk of bias, 4–6 stars indicating moderate risk of bias, and 7–9 stars indicating low risk of bias. Two reviewers independently performed the assessment. The mean score was calculated and rounded up to the nearest whole number for reporting. Discrepancies of more than two stars between reviewers were resolved through discussion.

For each results section, the distribution of studies across risk-of-bias categories was reported to inform the assessment of certainty of evidence for the corresponding outcome. Potential reporting bias was assessed descriptively by comparing the number of studies reporting significant and non-significant associations between DXA outcomes and fracture risk.

## 3 Results

### 3.1 Search

**Fig. 1:**
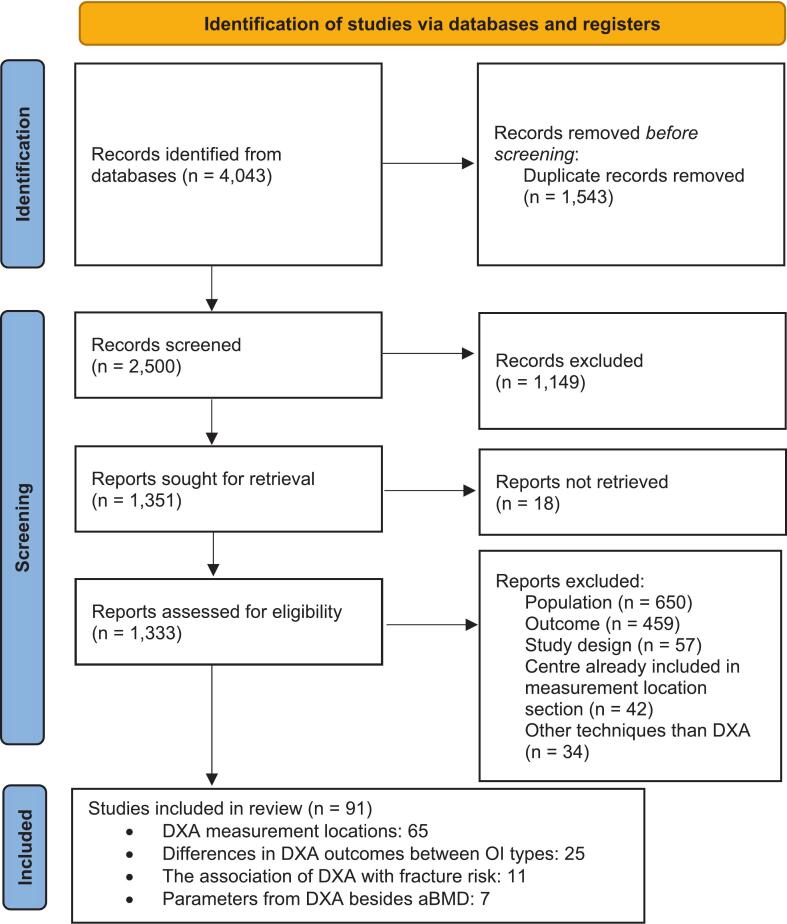
Flow diagram describing the inclusion process. Some studies were used in more than one subsection, so the numbers per section add up to more than the total number of included studies.

After removal of duplicates, 2501 articles from three databases were screened and 91 studies were included (Fig. 1). Quantitative data were extracted from 38 publications presenting results on 3696 patients.

### 3.2 DXA measurement locations

**Fig. 2:**
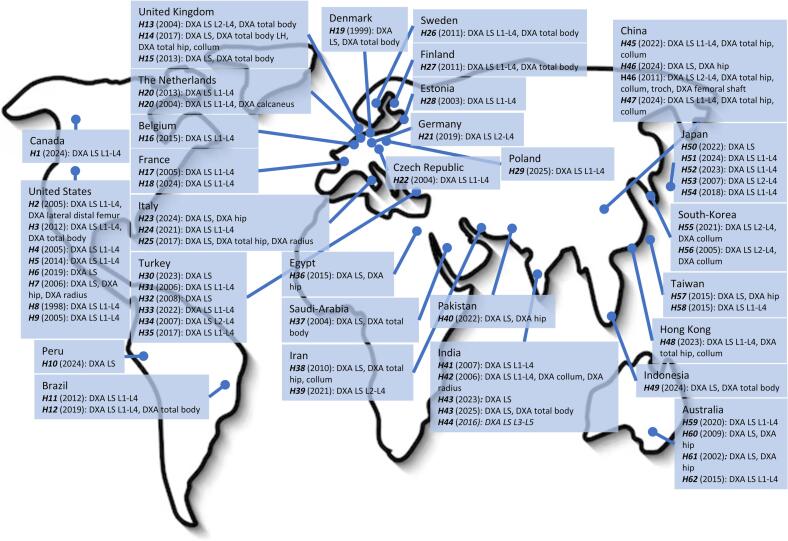
*Geographical distribution of research centres and DXA measurement sites assessed in paediatric patients with osteogenesis imperfecta.* LS = lumbar spine, L1-L5 indicate the vertebrae from proximal to distal. LH = less head (referring to the total body site), troch = trochanter (referring to the hip site). The exact location is specified if mentioned in the publication. If not, only the area (LS, hip) is described.

All 62 centres that published DXA results in a paediatric OI population used the lumbar spine as one of the regions of interest (Fig. 2, Table 1). Thirty of the centres added another measurement location; 11 of those used the total body scan, 20 measured at the proximal femur location, three the radius and one centre used either the femoral shaft, the lateral distal femur, or the calcaneus.

**Table 1:** For each hospital number (HX) from the map (Fig. 2), the respective research centre is listed together with the country and the used literature reference.

Nr	Country	Centre and literature reference	Nr	Country	Centre and literature reference
H1	Canada	Shriners Hospital for Children, Montreal (Glorieux et al., 2024)	H32	Turkey	Hacettepe University, Ankara (Andiran et al., 2008)
H2	US	Alfred I. duPont Hospital for Children, Wilmington (Grissom et al., 2005)	H33	Turkey	Istanbul University Faculty of Medicine (Öztürk et al., 2022)
H3	US	Indiana University School of Medicine, Indianapolis (Barros et al., 2012)	H34	Turkey	Marmara University School of Medicine, Istanbul (Akcay et al., 2008)
H4	US	National Institute of Child Health and Human Development, Maryland (Letocha et al., 2005)	H35	Turkey	Uludağ University Faculty of Medicine, Bursa (Aslan et al., 2017)
H5	US	Nationwide Children's Hospital, Columbus (Kusumi et al., 2015)	H36	Egypt	National Research Centre Cairo (Otaify et al., 2016)
H6	US	Shriners Hospital for Children, Chicago (Tayne and Smith, 2019)	H37	Saudi-Arabia	King Faisal Specialist Hospital and Research Centre, Riyadh (Bin-Abbas et al., 2004)
H7	US	Shriners Hospital for Children, Houston (Huang et al., 2006)	H38	Iran	Mofid Children ‘s Hospital, Shaheed Beheshti (Salehpour and Tavakkoli, 2010)
H8	US	Shriners Hospital for Children, St. Louis (Reinus et al., 1998)	H39	Iran	Shiraz University of Medical Sciences (Mohsenzade et al., 2021)
H9	US	Texas Scottish Rite Hospital for Children, Dallas (Seikaly et al., 2005)	H40	Pakistan	National Institute of Child Health, Karachi (Riaz et al., 2022)
H10	Peru	Instituto Nacional de Salud del Niño de Breña, Lima (Barba et al., 2024)	H41	India	All India Institute Of Medical Science, New Dehli (Bajpai et al., 2007)
H11	Brazil	Federal University of São Paulo (Chagas et al., 2012)	H42	India	B. J. Wadia Hospital for Children, Mumbai (Shah and Johari, 2007)
H12	Brazil	University of Rio Grande do Sul, Porto Alegre (Zambrano et al., 2019)	H43	India	Christian Medical College, Vellore (Selina et al., 2025, Selina et al., 2023)
H13	UK	The James Cook University Hospital Middlesbrough (Crabtree et al., 2004)	H44	India	Post Graduate Institute of Medical Education and Research, Chandigarh (Kumar et al., 2016)
H14	UK	Birmingham Children's Hospital (Saraff et al., 2018)	H45	China	Jiao Tong University Sixth People's Hospital, Shanghai (Zhang et al., 2022)
H15	UK	Sheffied Children's hospital (Bishop et al., 2013)	H46	China	Peking Union Medical College Hospital, Beijing (Li et al., 2011; Liu et al., 2024)
H16	Belgium	Cliniques Universitaires Saint-Luc, Brussels (De Wouters et al., 2022)	H47	China	University School of Medicine, Shanghai (Jiang et al., 2024)
H17	France	Hôpital d'enfants Armand Trousseau, Paris (Forin et al., 2005)	H48	Hong Kong	University of Hong-Kong-Shengzhen (Chen et al., 2023)
H18	France	Necker Enfants Malades teaching hospital, Paris (Charpié et al., 2024)	H49	Indonesia	Dr. Soetomo General Hospital Surabaya (Martanto et al., 2024)
H19	Denmark	Rigshospitalet Copenhagen (Lund et al., 1999)	H50	Japan	Aichi Children's Health and Medical Center, Obu (Sawamura et al., 2022)
H20	Netherlands	Wilhelmina Kinderziekenhuis Utrecht (Kok et al., 2013; Sakkers et al., 2004)	H51	Japan	Okayama University Hospital (Futagawa et al., 2024)
H21	Germany	University of Cologne (Hoyer-Kuhn et al., 2019)	H52	Japan	Osaka University Hospital (Ohata et al., 2023)
H22	Czech Republic	Charles University Hospital, Pilsen (Vyskočil et al., 2005)	H53	Japan	Sapporo Medical University School of Medicine (Watanabe et al., 2007)
H23	Italy	IRCCS Instituto Ortopedico Rozzoli, Bologna (Mordenti et al., 2023)	H54	Japan	Tohoku University hospital, Sendai (Kanno et al., 2018)
H24	Italy	University of Rome La Sapienza (Diacinti et al., 2021)	H55	South-Korea	Asan Medical Center, Seoul (Choi et al., 2022)
H25	Italy	University of Verona (Idolazzi et al., 2017)	H56	South-Korea	Seoul National University Children's Hospital (Cho et al., 2005)
H26	Sweden	Astrid Lindgren Children's Hospital, Karolinska (Heino et al., 2011)	H57	Taiwan	Chang Gung Memorial Hospital, Taipei (Kuo et al., 2015)
H27	Finland	Helsinki University Central Hospital (Vuorimies et al., 2011)	H58	Taiwan	Mackay Memorial Hospital, Taipei (Lin et al., 2015)
H28	Estonia	Tartu University Hospital (Maasalu et al., 2003)	H59	Australia	Royal Children's Hospital, Melbourne (Lim et al., 2021)
H29	Poland	Medical University of Lodz, Sporna (Nowicki and Jakubowska-Pietkiewicz, 2025)	H60	Australia	Monash University, Melbourne (Brown and Zacharin, 2009)
H30	Turkey	Ankara University Faculty of Medicine (Çetin et al., 2023)	H61	Australia	Royal Children's Hospital, Parkville (Zacharin and Bateman, 2002)
H31	Turkey	Ege University Faculty of Medicine Izmir (Göksen et al., 2006)	H62	Australia	Westmead Children's hospital (Biggin et al., 2015)

### 3.3 Differences in DXA outcomes between OI types

Twenty-three studies reported lumbar spine *Z*-scores in patients with varying types of OI, of which the BP treatment status varied (Table 2). Of these, ten studies were classified as having moderate risk of bias and 13 as having low risk of bias. The mean unweighted *Z*-score of type I patients over all studies was −2.3 (treated −2.0, untreated −2.4). Type III patients had a mean Z-score of −4.5 (treated −3.7, untreated −5.2) and type IV patients −3.2 (treated −2.4, untreated −3.5). The mean Z-score over the type V patients from three reporting studies was −2.1 (Bains et al., 2019; Öztürk et al., 2022; Valeeva et al., 2025). Most studies performing statistical testing on the data were consistently able to detect significant inter-group differences.

**Table 2:** Studies comparing DXA lumbar spine Z-scores between two or more OI types. NR = not reported. The column ‘significance’ indicates a significant statistical difference between any of the groups by ‘Y' = yes. No difference is indicated by ‘N' = no and ‘UNK’ (unknown) means that no statistical testing was performed. *only median value reported. Risk of bias was assessed using the Newcastle-Ottawa scale and resulted in a score of 1 (high risk) to 9 stars (low risk).

	Author	Year	N	Mean age	Type I	Type III	Type IV	Significance	Risk of bias
**BP**	Bains	2019	601	NR	–	−2.9	−2	UNK	7
	Florez	2022	31	40.5	−2.0	−3.5		N	5
	Futagawa	2024	29	5.4	−2.2	−2.2		N	6
	Kanno	2017	21	11.4	−3.5	−5.6	−3.2	Y	7
	Kashii	2019	34	8.7	−0.9	−3.9	−2.4	Y	6
	Nowicki	2025	61	8.0	−0.4	−3.14	–	Y	4
	Ozturk	2022	83	2.9	−3.2	−4.9	−1.1	Y	6
	Palomo	2015	37	14.5	–	−3.2	−2.2	UNK	8
**No BP**	Bains	2019	55	NR	–	−4	−2.5	UNK	7
	Kusumi	2015	18	0	−3.6	−4.8	−2.3	N	7
	Lindahl	2015	102	3.4	−2.4	−5.0	−4.2	UNK	8
	Lindahl	2016	79	6.8	−3.5	−5.3	−4.6	Y	7
	Liu	2017	103	NR	−0.8	−4	−2.6	Y	7
	Liu	2025	146	10.2	−0.6	−2.7	−1.6	Y	7
	Lund	1999	24	10	−4.2	−11.4	−5.6	Y	7
	Palomo	2015	37	14.3	–	−7.1	−5.8	UNK	8
	Song	2019	138	10	−2.0	−3.5	−3	Y	6
	Wang	2024	62	9.5	−0.9	−2.3	−2.3	Y	7
	Yazan	2021	65	3.5*	−3.7	−5	−4.3	UNK	6
	Scheres	2018	151	NR	−2.0	−4.1	−2.3	UNK	6
	Wekre	2011	52	44	−1.9	–	−3.2	Y	7
	Zheng	2022	225	8	−1.5	−3.4	−2.6	Y	7
	Ohata	2019	39	8	−2.3	−3.5	−3.4	N	7
**NR**	Li	2019	161	10.3	−1.6	−3.3	−3.2	Y	6
	Chagas	2012	26	25	−2.7	−2.7	–	N	5

DXA results from other measurement locations including the femoral neck, total hip and whole body were reported by 12 studies. Six of these studies were classified as having moderate risk of bias and six as having low risk of bias. The differences between the groups were similar to the lumbar spine location, with an unweighted mean of −1.9 for type I, −3.6 for type III, and − 2.6 for type IV (Table 3). This contributes to the high certainty of evidence for this section.

**Table 3:** Studies comparing DXA Z-scores at other locations apart from lumbar spine between two or more OI types. NR = not reported. The column ‘significance’ indicates a significant statistical difference between any of the groups by ‘Y' = yes. No difference is indicated by ‘N' = no. UNK = unknown. Risk of bias was assessed using the Newcastle-Ottawa scale and resulted in a score of 1 (high risk) to 9 stars (low risk).

	Author	Year	N	Mean age	BP	Type I	Type III	Type IV	Significance	Risk of bias
**Femoral neck**	Florez	2022	31	40.5	Yes	−1.5	−2.0		N	5
	Liu	2017	103	NR	No	−1.9	−6.1	−4.4	Y	7
	Liu	2025	146	10.2	No	−2.4	−4.6	−3.7	Y	7
	Song	2019	138	10	No	−2.7	−3.9	−3.3	Y	6
	Wang	2024	62	9.5	No	−2.2	−4.0	−3.5	Y	7
**Total hip**	Li	2019	161	10.3	UNK	−2.3	−4.5	−4.4	Y	6
	Florez	2022	31	40.5	Yes	−1.3	−0.7		N	5
	Scheres	2018	151	NR	No	−1.0	−2.3	−1.3	UNK	6
	Wekre	2011	68	44	No	−1.6	–	−1.1	N	7
	Zheng	2022	225	8	No	−2.7	−5.4	−4.1	Y	7
**Whole body**	Wekre	2011	90	44	No	−0.3	−1.3	−0.5	Y	7
	Lund	1999	24	10	No	−2.1	−4.1	−2.5	Y	7
**UNK**	Engelbert	2003	53	11.1	No	−3.4	−5.7	−4.3	UNK	5
	Valeeva	2025	45	30.8	UNK	−0.8	−2.2	−0.6	UNK	5

### 3.4 The association of DXA with fracture risk

Eleven studies reported the association between DXA results and fracture endpoints in OI patients. One of the studies was classified as having high risk of bias, four as having moderate risk, and 6 as having low risk of bias (Table 5). The number of publications reporting an association between DXA and fractures was similar to the number of publications reporting no association, suggesting no clear publication bias. The characteristics of the included patients, measurement locations, and fracture endpoints varied across studies (Table 4), limiting the certainty of evidence and partially explaining the heterogeneity among study results.

**Table 4:** Characteristics of the included studies reporting the association between areal bone mineral density (aBMD) or *Z*-score from and a fracture endpoint, categorized in the type of fractures assessed. UNK = unknown, BP = bisphosphonates, FU = follow-up.

	Author	Year	Type of study	N	Age group	Mean age	Clinical type	Treatment	DXA parameter	DXA location	Fracture endpoint
**All fractures**	Patel	2015	Cross-sectional	544	Mixed	12.6 (median)	I,III,IV	66% BP	aBMD	Lumbar spine	Incidence in preceding year
	Bains	2019	Prospective	256	Children	UNK	I,III,IV	2 groups: BP versus None	aBMD	Lumbar spine	Probability during FU (mean 3 years)
	Sun	2022	Prospective	201	Children	6.7	I,III,IV	BP	*Z*-score	Lumbar spine	Incidence during BP treatment ≥1 year
	Barba	2024	Retrospective	91	Children	UNK	NR	75% BP	*Z*-score	Lumbar spine	Lifetime number
	Wekre	2011	Cross-sectional	90	Adults	44	I,III,IV	18% BP	aBMD	Total body	Lifetime incidence
	Blandin	2024	Prospective	71	Adults	41.4	I,IV	6% BP	*Z*-score	Lumbar spine/hip/radius	Incidence per FU time (median 5 years)
	Koumakis	2022	Retrospective	50	Adults	29.7	I,III,IV	None	*Z*-score	Lumbar spine/hip	Yes/no during pregnancy
	Robinson	2019	Retrospective	31	Mixed	16.4	I,III,IV	BP	Z-score	Lumbar spine	Yes/no in 4 years since BP stop
	Huang	2006	Retrospective	20	Children	UNK	I,III,IV	None	aBMD	Lumbar spine	Lifetime rate
**Vertebral fractures**	Ben Amor	2013	Retrospective	58	Children	7.4	I	None	Z-score	Lumbar spine	Yes/no on X-ray
	Reinus	1998	Retrospective	27	Children	11.7	I,III,IV	None	aBMD, Z-score	Lumbar spine	Lifetime rate
**Long bone fractures**	Ben Amor	2013	Retrospective longitudinal	48	Children	3.6	I	None	Z-score	Lumbar spine	Annual rate during ≥2 years FU

Across the included studies, two focused specifically on either long-bone or vertebral fractures, whereas most examined fractures of any type (Table 4). The corresponding findings are summarized in Table 5. Ben Amor et al. found no significant association between baseline aBMD and long-bone fracture incidence during two years of follow-up (Ben Amor et al., 2013). In contrast, both studies focussing on vertebral outcomes reported a significant relationship between lower aBMD and the presence of vertebral compression fractures on imaging (Ben Amor et al., 2013; Reinus et al., 1998).

**Table 5:** Outcomes of the included studies reporting the association between aBMD or *Z*-score from DXA and a fracture endpoint, categorized by the type of fractures assessed. In case of a regression analysis, the Single/Multi-variable column shows whether only one or several parameters were included in the analysis. In case of multi-variable analysis, the effect of the DXA outcome is assessed while all other variables are kept constant. The arterisk (*) indicates that the authors reported a significant effect. LS = lumbar spine, TB = total body, NR = not reported, BP = bisphosphonates, FU = follow-up. Risk of bias was assessed using the Newcastle-Ottawa scale and resulted in a score of 1 (high risk) to 9 stars (low risk).

	Author	DXA outcome	Fracture endpoint	Statistical method	Single/Multi-variable	Effect measure	Effect size	Risk of bias
**All fractures**	Patel	LS aBMD	Incidence in preceding year	Correlation	–	Correlation coefficient	I: 0.02, III: 0.05, IV: −0.02	5
	Bains	LS aBMD	Probability during FU (mean 3 years)	Logistic regression	Single	Percentage change	BP: −24.3*, None: 19.4	7
	Bains	LS aBMD	Probability during FU (mean 3 years)	Logistic regression	Multi	Regression coefficient	0.81	7
	Sun	LS *Z*-score	Incidence during BP treatment ≥1 year	Logistic regression	Single	Odds ratio	1.18	6
	Barba	LS *Z*-score	Lifetime number	Linear regression	Single	Regression coefficient	−0.213*	3
	Wekre	TB aBMD	Lifetime number	Linear regression	Multi	NR	NR*	7
	Blandin	*Z*-score < −2 vs ≥ −2	Incidence during FU (median 5 years)	Logistic regression	Multi	Odds ratio	4.38*	7
	Koumakis	*Z*-score	Yes/no during pregnancy	MWU test	–	Difference in Z-score	LS: −2.9 (yes), −1.5 (no)*, hip: −2.0 (yes), −0.5 (no)*	7
	Robinson	LS Z-score	Yes/no in 4 years since BP stop	*t*-test	–	Difference in Z-score	−2.0 (yes), −1.7 (no)	7
	Huang	LS aBMD	Lifetime rate	Pearson correlation	–	Correlation coefficient	−0.69*	4
**Vertebral fractures**	Ben Amor	LS Z-score	Yes/no on X-ray	Logistic regression	Multi	Odds ratio	0.4*	8
	Reinus	LS aBMD, Z-score	Lifetime rate	Correlation	–	Correlation coefficient	aBMD: −0.47*, Z-score: −0.6*	6
**Long bone fractures**	Ben Amor	LS Z-score	Annual rate during ≥2 years FU	Logistic regression	Multi	Odds ratio	NR	8

Nine studies investigated the association between DXA results and overall fracture risk using diverse methodological approaches (Table 4). Five reported significant associations (Barba et al., 2024; Blandin et al., 2024; Huang et al., 2006; Koumakis et al., 2022; Wekre et al., 2011); notably, all three studies conducted exclusively in adult populations identified a relationship between lower aBMD and increased fracture risk (Blandin et al., 2024; Koumakis et al., 2022; Wekre et al., 2011). However, three studies did not observe any association between DXA results and fracture risk (Patel et al., 2015; Robinson et al., 2019; Sun et al., 2022).

The ninth study by Bains et al., who followed 256 OI patients below 14 years longitudinally with yearly assessments, reported varying results depending on analytic approach and treatment status (Bains et al., 2019). In BP-treated children, higher aBMD values were associated with reduced fracture probability, whereas no association was observed in untreated children. In a multivariable model including aBMD, age, and BP use in the full cohort (Bains et al., 2019), increase of aBMD was not independently associated with decreased fracture probability.

### 3.5 Additional DXA parameters

Bone parameters derived from DXA beyond aBMD are described in this section, together with the available results in OI patients (see overview in Table 6). Overall, the evidence for the five DXA-based parameters was scarce. Seven studies reported results in OI patients, of which one was classified as high risk of bias, four as moderate risk, and two as low risk.

**Table 6:** Overview of parameters derived from DXA besides BMD and studies including these. Risk of bias was assessed using the Newcastle-Ottawa scale and resulted in a score of 1 (high risk) to 9 stars (low risk).

DXA-derived parameter	Author	Year	N	Risk of bias
Trabecular bone score (TBS)	Florez	2022	31	5
	Futagawa	2024	21	6
	Kocijan	2015	30	5
	Liu	2025	153	7
	Ohata	2023	42	7
Hip structure analysis (HSA)	Kocijan	2013	30	6
Bone mineral apparent density (BMAD)	Mohsenzade	2021	23	3
	Ohata	2023	42	7
Bone mineral height-adjusted *Z*-score (BMD_HAZ_)	Ohata	2023	42	7
Dual energy X-ray laser absorptiometry (DXL)	Kocijan	2015	30	5

#### 3.5.1 Correcting aBMD for size

To distinguish whether low DXA-derived density values reflect intrinsically abnormal bone tissue properties rather than simply reduced bone size, several studies have proposed adjusting aBMD for size. In order to correct for the generally short stature of OI patients, a height-for-age Z-score (HAZ)-adjusted aBMD Z-score (BMD_HAZ_) can be calculated (Zemel et al., 2010). This was found to be not predictive of fracture risk in a study including OI patients (Ohata et al., 2023).

A second approach is to estimate the volume of the bone and correct aBMD for this, yielding a volumetric density estimate, which is commonly reported as bone mineral apparent density (BMAD) or estimated vBMD (Carter et al., 1992; Kröger et al., 1992). For consistency throughout this review, these derived volumetric measures will be referred to collectively as BMAD.

Several studies have adopted the volume correction method originally described by Carter (Brown and Zacharin, 2009; Mohsenzade et al., 2021; Ohata et al., 2023; Rauch et al., 2006a, Rauch et al., 2006b, Rauch et al., 2003; Zeitlin et al., 2006). This method models the vertebral bodies as a cube and calculates BMAD using the following equation: BMAD = BMC/(projection area)^1.5^, with unit g/cm^3^ (Carter et al., 1992).

Two studies evaluated the relationship between the BMAD according to Carter and fracture outcomes. In a cross-sectional study of 42 patients between 5 and 20 years old (Ohata et al., 2023), lumbar spine BMAD was estimated and BMAD Z-scores were calculated using reference values (Kindler et al., 2019). In the total OI cohort, BMAD Z-scores were not correlated to lifetime yearly fracture rate. However, when analyses were stratified by underlying type of mutation, a negative correlation was found between BMAD Z-score and fracture rate in the – generally more severely affected – glycine substitution group (*r* = −0.74, *p* = 0.022), whereas no such correlation was present in the haploinsufficiency group. A second study assessed BMAD as a predictor of fracture risk in a retrospective cohort of OI children receiving BP therapy (Mohsenzade et al., 2021). In this population, baseline lumbar spine BMAD did not significantly predict fracture rate during treatment.

Kröger et al. have introduced a similar method to correct DXA outcomes for bone volume. In their method, the lumbar vertebrae are modelled as a cylinder and BMAD is calculated using the following equation: BMAD = aBMD * [4/(π x projection width)] (Kröger et al., 1993, Kröger et al., 1992). Two studies applied this method to OI patients undergoing BP treatment, but did not investigate the association with OI type or fracture risk, and were therefore not included (Bishop et al., 2010; Vyskočil et al., 2005).

#### 3.5.2 Dual X-ray and laser (DXL)

To correct for soft tissue attenuation, the DXA scanner uses two X-ray energies and calculates patient-specific mean soft tissue attenuation. However, soft tissue includes lean mass as well as fat, which have varying attenuation factors and are non-homogeneously distributed (Joyet et al., 1974). Therefore, soft tissue differences can induce significant aBMD measurement errors. The method called DXL was introduced to combine calcaneal DXA with a heel thickness measurement via laser detection, which results in an aBMD measurement corrected for the lean-to-fat ratio (Hakulinen et al., 2003). In a cohort of 30 adults with OI, DXL resulted in lower aBMD in OI type III/IV patients as compared to type I (Kocijan et al., 2015). Using conventional DXA at the lumbar spine, no difference was observed between these OI types. The association with fracture endpoints was not assessed.

#### 3.5.3 Trabecular bone score (TBS)

TBS quantifies the texture of the lumbar spine DXA image by evaluating the variation in pixel intensities within the projected trabecular bone area. The underlying premise is that a well-connected, dense trabecular network produces greater local grey-level variability, whereas degraded or sparse trabeculae result in more homogeneous pixel patterns. The predictive value for fracture incidence has been proven in non-OI women (Boutroy et al., 2013). Two studies assessed the influence of treatment on TBS, but did not investigate the relation with OI type or fractures (Lin et al., 2024; Rehberg et al., 2019).

Four studies compared TBS between clinical types of OI, and none of those found a significant difference (Florez et al., 2022; Futagawa et al., 2024; Kocijan et al., 2015; Liu et al., 2025). Since high-resolution peripheral quantitative computed tomography (HR-pQCT) is generally considered the best available method to assess microarchitectural parameters, the association between TBS and trabecular outcomes of HR-pQCT was investigated (Kocijan et al., 2015). However, there was no significant association between TBS and HR-pQCT-derived microstructure.

Two studies investigated fracture risk prediction by means of TBS. In a cohort of 42 children and young adults with OI, no correlation between TBS and lifetime fracture rate was observed. In an analysis stratified on genetic mutation, lower TBS was correlated with higher fracture risk (*r* = −0.5, *p* = 0.042) in patients with a haploinsufficient defect mutation, who are generally less severely affected than patients with a glycine substitution mutation (Ohata et al., 2023). To discriminate between patients with and without vertebral compression fractures, combining aBMD measurement with TBS was equally effective as aBMD alone (Liu et al., 2025).

#### 3.5.4 Hip structure analysis (HSA)

Hip Structure Analysis (HSA) aims to derive geometric parameters from the DXA hip to more accurately estimate bone strength (Beck et al., 1990). While the cross-sectional area (CSA) shows the area of the bone at a cross-section perpendicular to the shaft, the cross-sectional moment of inertia (CSMI) indicates the distribution of bone mass around the centre of the bone at that cross-section. Assuming a constant volumetric tissue density, CSA can be derived by integrating over the X-ray absorption curves of that cross-section (Beck et al., 1990; Martin and Burr, 1984). CSMI results from multiplying the pixel intensities by their distance from the centre of the neck and summing these for each cross-section. Using the CSA and CSMI, the compressive strength of the bone can be calculated. The femoral strength index (FSI) is the ratio of this compressive strength to the estimated stress resulting from a fall on the greater trochanter (Faulkner et al., 2006).

These three parameters were compared between clinical types of adult OI patients and controls (Kocijan et al., 2013). CSA was significantly reduced in OI type III/IV compared to type I, and in type I compared to controls. CSMI was lower in OI than in controls, but did not differ between types of OI. The FSI was decreased in OI type III/IV as compared to controls, but no differences were reported between the other groups. Although the association with fracture incidence was not established in this study, the authors believe that especially CSA and CSMI could be a valuable parameter for fracture risk estimation in OI.

## 4 Discussion

This systematic review aimed to provide an overview of fracture risk assessment in children with OI, focussing on current clinical practice using DXA-derived aBMD and alternative DXA-based methods.

As DXA is a two-dimensional technique that cannot differentiate between trabecular and cortical compartments, the interpretation of results is strongly influenced by the anatomical site measured. Lumbar spine measurements predominantly capture trabecular bone density, whereas total body assessments especially reflect cortical bone property (Messina et al., 2018). Hip measurements represent a composite of both compartments, but in young children they are unreliable because the proximal femur is not yet fully ossified (International Society For Clinical Densitometry, 2019; Messina et al., 2018).

Across centres publishing DXA research in children with OI, lumbar spine measurements were universally reported, though the exact region varied between L1–L4, L2–L4, and L3-L5. Selecting L2 or L3 as the starting vertebra may reflect concerns that the upper lumbar vertebrae are more prone to compression fractures (artificially increasing density despite underlying fragility), and potential overprojection due to deformities. Although the International Society for Clinical Densitometry recommends total body scans for DXA assessment in all children, only 10 out of 61 centres have reported utilizing this method in publications involving children with OI. This limited adoption might be attributable to the technique's sensitivity to metal implants and bone deformities, which may constrain the applicability in OI research. Possibly, the total body DXA is more frequently used for clinical longitudinal monitoring of individual patients than for published research purposes.

The results showed that patients with moderate to severe OI have lower aBMD compared to patients with mild OI. In general, more disease severity is associated with higher fracture risk, which suggests that the Z-scores derived from DXA measurement may have predictive value for fracture risk. However, this observation should be interpreted with caution. DXA cannot distinguish between true volumetric bone density and bone size, and individuals with more severe OI are characteristically shorter with smaller bones. As a result, reductions in aBMD Z-scores may partially reflect differences in bone geometry rather than intrinsic bone tissue properties.

The relationship between DXA aBMD and fracture risk in OI patients remains uncertain, with studies reporting inconsistent associations. Whether a meaningful correlation is detected seems to depend on the fracture outcome assessed, including its anatomic location and the timeframe considered. aBMD appears to relate more consistently to broad, cumulative fracture endpoints – such as lifetime fracture rate – than to more specific endpoints like recent fracture history or short-term fracture incidence. However, these latter outcomes are particularly relevant for treatment decision-making in patient follow-up. Moreover, some evidence suggests that DXA may better predict site-specific fracture risk, which suggests that measurement at the most fracture-prone locations could increase clinical utility.

These limitations of DXA aBMD have encouraged exploration of several alternative DXA-derived parameters, yet most have not proven clinically useful in the limited amount of literature available. Correcting aBMD for patient height (BMD_HAZ_) or estimated bone volume (BMAD) do not appear to support fracture risk prediction. TBS, a surrogate measure of cancellous bone architecture, failed to demonstrate predictive value in the OI population. This may be related to fact that in OI patients, diaphyseal fractures are most common with predominantly cortical bone, whereas TBS aims to capture trabecular texture. Similarly, the DXL technique, which corrects for soft-tissue-related inaccuracies by measurement of bone thickness by laser, does not overcome fundamental geometric limitations of DXA in OI.

In contrast, geometric approaches such as HSA, which is based on DXA hip, appear more promising. Although the femoral neck is not an ideal site for DXA assessment in children with OI, HSA's focus on bone geometry aligns well with the mechanical determinants of bone strength that are highly relevant in this population. Geometric assessment of diaphyseal regions, the primary fracture sites in OI, could therefore offer a more relevant strategy.

Given these results of current DXA-derived measures in the OI population, there is a need for alternative methods of bone quality assessment in patients with OI. However, clinically established alternatives for DXA assessment in OI are currently not available. (HR-)pQCT may offer opportunities for bone strength assessment in OI patients, as it enables three-dimensional assessment of cortical and trabecular bone compartments at peripheral long bone locations (Gazzotti et al., 2024). In a research context, the technique has demonstrated sensitivity to differences in tissue properties and geometry between OI patients and controls, as well between different OI types (Bartosik et al., 2025; Coussens et al., 2022; Folkestad et al., 2012; Gatti et al., 2003; Hald et al., 2016; Hosseinitabatabaei et al., 2025; Kocijan et al., 2015; Rolvien et al., 2018; Simon et al., 2022). However, more research is needed before this technique can find its way to clinical practice (Gazzotti et al., 2023).

### 4.1 Strengths and weaknesses

For this review, all papers resulting from our search term presenting information on DXA assessment in OI patients were included. This resulted in a complete overview of current clinical practice and research with DXA in OI. However, no meta-analysis was done using the data on the differences in DXA outcomes between OI types or the association of DXA with fracture risk, given the heterogeneity of the included publications. We do not believe that this impacts the message of this review.

## 5 Conclusion

DXA measurements at the lumbar spine remain the most commonly used tool to guide treatment in patients with OI. However, the association between DXA-derived aBMD and fracture risk is weak, especially if fracture incidence is assessed over shorter time intervals. Alternative DXA-based parameters have not demonstrated superior performance in assessing bone strength in OI, likely because most were originally developed for osteoporosis and insufficiently capture the mechanical determinants of fragility specific to OI. Given these results, there is a need for alternative methods of bone quality assessment in patients with OI. DXA assessment focussing on fracture-prone skeletal regions and incorporating geometric properties may strengthen the clinical value of DXA.

## CRediT authorship contribution statement

**Karlijn Scheepens:** Writing – original draft, Investigation, Conceptualization. **Suzanne den Haan:** Writing – review & editing, Investigation. **Kelly Warmink:** Data curation, Writing – review & editing. **Ralph Sakkers:** Writing – review & editing, Conceptualization. **Anne Spaans:** Writing – review & editing. **Wouter Nijhuis:** Conceptualization. **Harrie Weinans:** Writing – review & editing, Conceptualization.

## Other information

This review was not registered and no protocol was published. No funding has been received. All data have been included in this manuscript.

## Declaration of Generative AI and AI-assisted technologies in the writing process

During the preparation of this work the author(s) used Chat-GPT (GPT-5.3, OpenAI, San Francisco, CA, USA) in order to improve the language of the manuscript. After using this tool/service, the authors reviewed and edited the content as needed and take full responsibility for the content of the published article.

## Declaration of competing interest

The authors declare that they have no known competing financial interests or personal relationships that could have appeared to influence the work reported in this paper.

## Data Availability

No data was used for the research described in the article.
